# Effect of Bariatric Surgery on Intima Media Thickness: A Systematic Review and Meta-Analysis

**DOI:** 10.3390/jcm11206056

**Published:** 2022-10-13

**Authors:** Tannaz Jamialahmadi, Željko Reiner, Mona Alidadi, Wael Almahmeed, Prashant Kesharwani, Khalid Al-Rasadi, Ali H. Eid, Manfredi Rizzo, Amirhossein Sahebkar

**Affiliations:** 1 Applied Biomedical Research Center, Pharmaceutical Technology Institute, Mashhad University of Medical Sciences, Mashhad 9177948564, Iran; 2 Department of Nutrition, Faculty of Medicine, Mashhad University of Medical Sciences, Mashhad 9177918564, Iran; 3 Department of Internal Medicine, University Hospital Center Zagreb, 10000 Zagreb, Croatia; 4 Heart and Vascular Institute, Cleveland Clinic Abu Dhabi, Abu Dhabi P.O. Box 124140, United Arab Emirates; 5 Department of Pharmaceutics, School of Pharmaceutical Education and Research, Jamia Hamdard, New Delhi 110062, India; 6 Medical Research Centre, Sultan Qaboos University, Muscat P.O. Box 373, Oman; 7 Department of Basic Medical Sciences, QU Health, Qatar University, Doha P.O. Box 2713, Qatar; 8 Department of Health Promotion, Mother and Child Care, Internal Medicine and Medical Specialties, School of Medicine, University of Palermo, 90133 Palermo, Italy; 9 Biotechnology Research Center, Pharmaceutical Technology Institute, Mashhad University of Medical Sciences, Mashhad 9177948954, Iran; 10 Department of Biotechnology, School of Pharmacy, Mashhad University of Medical Sciences, Mashhad 9177948954, Iran

**Keywords:** obesity, bariatric surgery, intima-media thickness, atherosclerosis, meta-analysis, coronary heart disease

## Abstract

Background: Obesity, and in particular extreme obesity, as a global health problem is an important risk factor for many diseases, including atherosclerotic cardiovascular disease (ACVD). Bariatric surgery might stop or slow atherogenesis by decreasing excessive weight in the early stages of atherogenesis, by suppressing low-grade systemic inflammation as well as by inhibiting oxidative stress and endothelial dysfunction. The aim of this meta-analysis was to provide an answer to whether bariatric surgery has a significant effect on intima-media thickness (IMT) which is a surrogate marker of early atherosclerosis and has a good correlation with atherosclerotic coronary heart disease. Methods: A systematic literature search in PubMed, Scopus, Embase, and Web of Science as well as grey literature was performed from inception to 1 July 2022. The meta-analysis was performed using Comprehensive Meta-Analysis (CMA) V3 software. Overall, the estimate of effect size was measured by a random effects meta-analysis. To account for the heterogeneity of studies regarding study design, characteristics of the populations, and treatment duration, a random-effects model (using the DerSimonian–Laird method) and the generic inverse variance weighting approach were used. To assess the existence of publication bias in the meta-analysis, the funnel plot, Begg’s rank correlation, and Egger’s weighted regression tests were used. Results: The meta-analysis of 30 trials, including 1488 subjects, demonstrated a significant decrease in IMT after bariatric surgery. The reduction in IMT was also robust in the leave-one-out sensitivity analysis. It must be stressed that the results of the random-effects meta-regression did not suggest any relationship between the changes in IMT and delta body mass index (BMI) or duration of follow-up after the bariatric surgery. However, the subgroup analyses showed a better IMT reduction after laparoscopic sleeve gastrectomy (LSG) when compared to Roux-en-Y gastric bypass (RYGB). Within a year, the IMT follow-up values showed a further improvement. Conclusions: Bariatric surgery significantly reduced IMT. Significant associations were found between the surgery type and IMT changes, as well as a significant effect of follow-up duration on the changes of IMT after bariatric surgery.

## 1. Introduction

Almost all countries are witnessing a pandemic of overweight and obesity with a devastating trend, which is best illustrated by the fact that obesity has nearly tripled since 1975. In 2016, more than 1.9 billion adults aged 18 years and older were overweight and 3% of the world’s population, or more than 650 million people, were obese with an increasing prevalence [1]. In most developed countries, the rates of obesity are much higher so that, for example, in the USA more than 40% of adults are obese [2]. It is well known that obesity as a global health issue is also an important risk factor for many diseases, including atherosclerotic cardiovascular disease (ACVD), and it is associated with an increased ACVD morbidity and mortality [3]. This is primarily explained by a systemic low-grade inflammatory state in obesity which is not only a risk for ACVD but also for metabolic syndrome (MetSy), type 2 diabetes mellitus (T2DM), nonalcoholic fatty liver disease (NAFLD), nonalcoholic steatohepatitis (NASH), chronic kidney disease, different types of cancers, and other inflammatory diseases, including pancreatitis, psoriasis, atopic dermatitis, and autoimmune arthritis [4,5,6,7,8]. Obesity is also associated with oxidative stress which may promote the development of vascular wall lesions causing endothelial dysfunction; thus, predisposing the arterial wall to morphological and functional damages leading to atherogenesis.

No matter how it is achieved, weight reduction decreases the risk of ACVD, cardiovascular events, as well as cardiovascular and total mortality. Bariatric surgery is a surgical treatment, which is used primarily for patients who are severely obese to decrease their excessive weight. The types of bariatric surgery are sleeve gastrectomy (SG), laparoscopic adjustable gastric band (LAGB), Roux-en-Y gastric bypass (RYGB), biliopancreatic diversion/duodenal switch (BPD/DS), and one anastomosis gastric bypass/mini gastric bypass (OAGB/MGB) [9]. There are data suggesting the positive impact of bariatric surgery on several cardiometabolic indicators [10,11,12,13,14,15]. There have been reports indicating that bariatric surgery might prevent or slow down atherogenesis in the early stages by breaking the vicious circle between inflammation and endothelial dysfunction [16].

Measuring intima-media thickness (IMT), particularly carotid IMT (CIMT), by ultrasonography is considered to be a surrogate marker of early atherosclerotic changes in the arteries. This could help to improve the prediction of cardiovascular events in different arterial territories because of the positive correlation between increased IMT and atherosclerotic changes in coronary arteries, i.e., with coronary heart disease (CHD) [17,18,19,20]. Therefore, IMT is used in predicting CHD and improving the cardiovascular risk prediction models. Although some studies have suggested that bariatric surgery has a beneficial effect and might decrease IMT, other studies could not find any change in IMT in obese patients after bariatric surgery.

Following bariatric surgery, several cardiovascular-related risk factors can be improved, including insulin resistance, type 2 diabetes, hypertension, and hyperlipidemia; however, it is worth mentioning that these improvements are not the only effect of weight loss [21,22]. Since obesity provokes an inflammation-prone environment, bariatric surgery seems to decrease cytokines involved in this process, especially CRP and IL-6, as shown in a recent meta-analysis [23,24,25].

Since the data concerning the effects of bariatric surgery on IMT are conflicting, the aim of this systematic review and meta-analysis is to provide a clear answer as to whether bariatric surgery can decrease IMT or not.

## 2. Methods

### 2.1. Search Strategy

The 2009 preferred reporting items for systematic reviews and meta-analysis (PRISMA) guidelines were used to prepare this systematic review and meta-analysis [26]. PubMed, Scopus, Embase, Web of Science, as well as grey literature (CareSearch, Google, and the Grey Literature Report), and all reference lists of retrieved articles were searched from inception to 1 July 2022 using the following keywords in titles and abstracts: (“intima media thickness” OR “intima-media thickness” OR “carotid intima media thickness” OR “carotid intima media” OR “artery intima media thickness” OR “intima media thickness measurement” OR “intima media thickness cardiovascular” OR “carotid intima media thickness measurement” OR “intima-media thickness measurements” OR “carotid intima media thickness cardiovascular” OR “intima-media thickness” OR CIMT OR IMT OR “carotid intima-media thickness” OR “carotid intima media” OR “Carotid atherosclerosis” OR “intima-media”) AND (“bariatric surgery” OR gastroplast* OR “gastric bypass” OR “Roux-en-Y” OR “gastric band” OR “biliopancreatic diversion” OR gastrectom* OR “duodenal switch” OR “weight loss surgery” OR “gastrointestinal diversion” OR gastroenterostom* OR “jejunoileal bypass” OR “obesity surgery” OR “weight-loss surgery” OR “bariatric procedure” OR “sleeve surgery” OR “metabolic surgery”).

### 2.2. Study Selection

All studies investigating the effects of bariatric surgery on carotid intima media thickness (CIMT) were included, based upon our pre-determined inclusion criteria. Case studies, non-English studies, reviews, and animal studies were not considered. A study had to provide documented CIMT data before surgery and after a post-operative observation period to be included in this meta-analysis. This systematic review and meta-analysis was not registered in any registry.

### 2.3. Data Extraction

All titles and abstracts were separately screened by two authors (TJ and MA). When there was a disagreement concerning the eligibility of a study, the paper was examined collaboratively, and a decision was reached. Study characteristics (the name of the primary author, the year of publication, study design, type of surgery, length of follow-up, health status of the participants, major clinical and demographic variables, values of IMT, and sample size) were extracted from each study.

### 2.4. Quality Assessment

The Newcastle–Ottawa Scale (NOS) was used to estimate the quality of the studies included in this meta-analysis [27,28]. This scale considers three features of each qualified study: (1) study patient selection (4 elements); (2) study population comparability (one item); and (3) exposure determination (3 items) in case-control studies or result of interest in cohort studies.

### 2.5. Quantitative Data Synthesis

The meta-analysis was performed using Comprehensive Meta-Analysis (CMA) V3 software (Biostat Inc., Englewood, NJ, USA) [29]. The weighted mean difference (WMD) with relevant CIs was determined for continuous outcomes. From each group, sample sizes, means, and standard deviations were obtained for each relevant outcome to calculate WMD. Overall, the estimate of effect size was measured by a random effects meta-analysis. To account for the heterogeneity of studies with regard to study design, characteristics of the populations, and treatment duration, the random-effects model (using the DerSimonian–Laird method) and the generic inverse variance weighting approach were used [26]. Sensitivity analysis using the leave-one-out approach (i.e., deleting one study each time and repeating the analysis) was applied to analyze the effect of each study on the overall effect size [30].

### 2.6. Meta-Regression

To investigate the association between BMI change and follow-up duration after surgery with the estimated effect size, these parameters were included into a random-effect meta-regression model.

### 2.7. Subgroup Analysis

A subgroup analysis was completed to describe heterogeneity, and to further characterize outcomes for the type of surgery and follow-up period.

### 2.8. Publication Bias

The funnel plot, Egger’s weighted regression, as well as Begg’s rank correlation tests were used to examine the presence of publication bias in the meta-analysis. The “trim and fill” approach was used to insert potentially missing studies when there were indications of funnel plot asymmetry. In the case of a significant result, the number of potentially missing studies needed to make the *p*-value non-significant was determined using the “fail-safe N” approach as another evidence of publication bias [31].

## 3. Results

**Figure 1:**
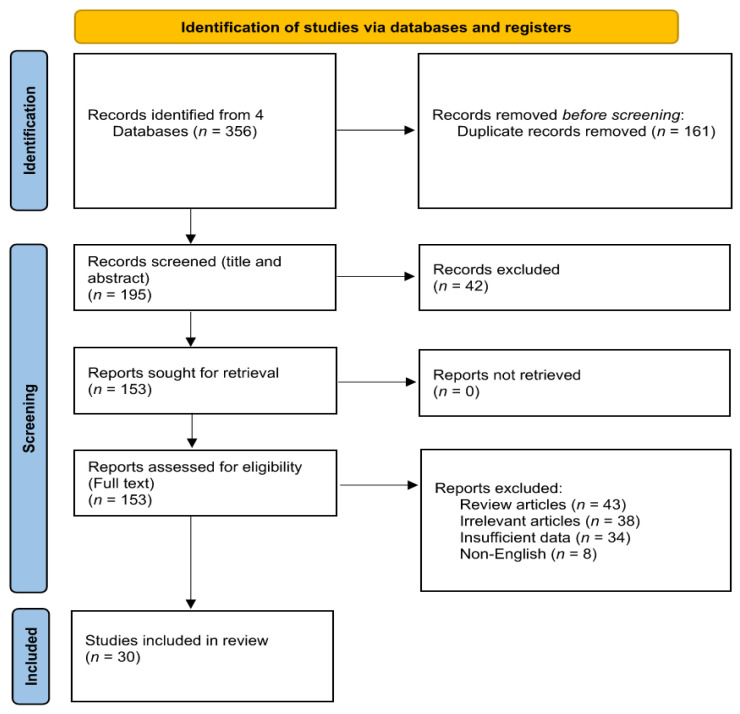
Flowchart of the included studie.

Among 356 published studies identified by a systematic databases search, 173 were directly related to the topic of this study. In total, 143 studies were excluded after careful evaluation (43 studies were reviews, 38 studies were excluded because they did not match the inclusion criteria, 34 studies did not report sufficient data, and 8 were non-English papers). Therefore, 30 studies which evaluated IMT after bariatric surgery were included (Table 1). Figure 1 shows the process of study selection.

### 3.1. Quality Assessment of the Included Studies

In cohort studies, although most of the selected studies [16,32,33,35,36,37,38,39,40,41,42,43,44,45,46,48,49,50,51,52,53,54,55,56,57,58,59,60] showed representativeness of the cases, the majority of them were distinguished by a lack of nonexposed group definition information. Since most of the studies did not include a control group, they were not assessed for comparability. In case-control studies [34,47], the included studies met the selection and exposure criteria. Quality assessment of the selected studies is presented in Table 2 and Table 3.

### 3.2. Effect of Bariatric Surgery on IMT

**Figure 2:**
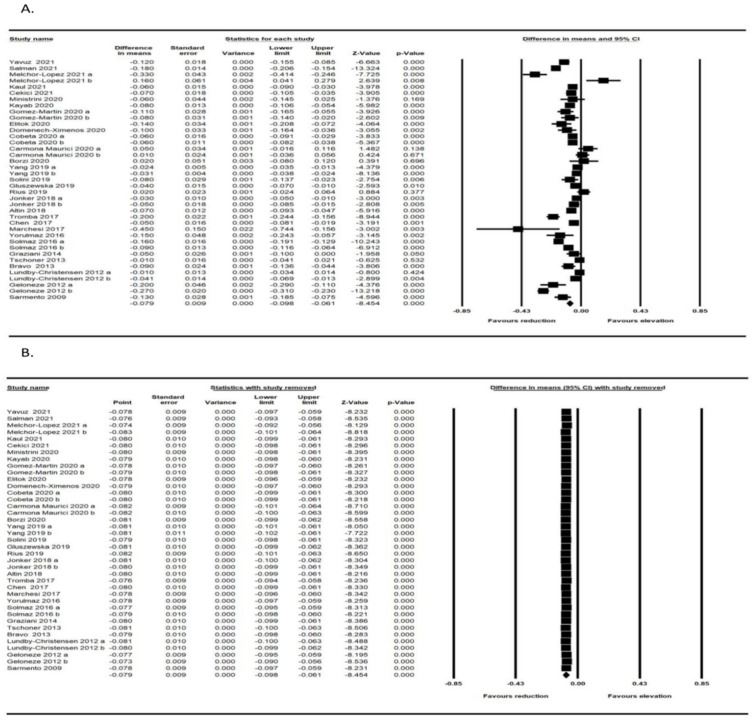
(**A**) Forest plot displaying standardized mean difference and 95% confidence intervals showing the consequence of bariatric surgery on IMT [16,32,33,34,35,36,37,38,39,40,41,42,43,44,45,46,47,48,49,50,51,52,53,54,55,56,57,58,59,60]; (**B**) Leave-one-out sensitivity analyses indicating the effect of bariatric surgery on IMT [16,32,33,34,35,36,37,38,39,40,41,42,43,44,45,46,47,48,49,50,51,52,53,54,55,56,57,58,59,60].

The meta-analysis of 30 trials, including 1488 subjects, demonstrated a significant decrease in IMT after bariatric surgery (WMD: −0.081, 95% CI: −0.101, −0.061, *p* < 0.001) (Figure 2A). The reduction in IMT was robust in the leave-one-out sensitivity analysis (Figure 2B). In other words, the iterative removal of each included trial from the meta-analysis did not cause a significant change in the pooled estimate of effect size.

### 3.3. Meta-Regression

**Figure 3:**
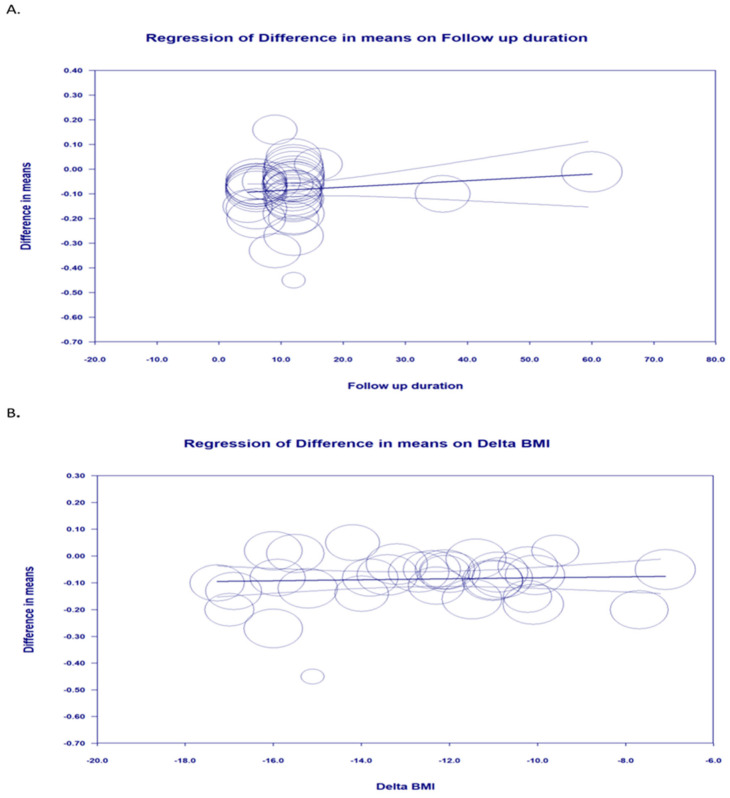
Random-effects meta-regression for evaluating the effect of: (**A**) delta BMI; (**B**) follow-up duration.

The impact of potential confounders on the IMT reducing the effect of bariatric surgery was assessed by random-effects meta-regression. The findings did not suggest any relationship between the changes in IMT and delta body mass index (BMI) (slope: 0.002; 95% CI: −0.005, 0.010; *p* = 0.670) or duration of follow-up (slope: 0.001; 95% CI: −0.0008, 0.0034; *p* = 0.227) (Figure 3A,B).

### 3.4. Subgroup Analysis

**Figure 4:**
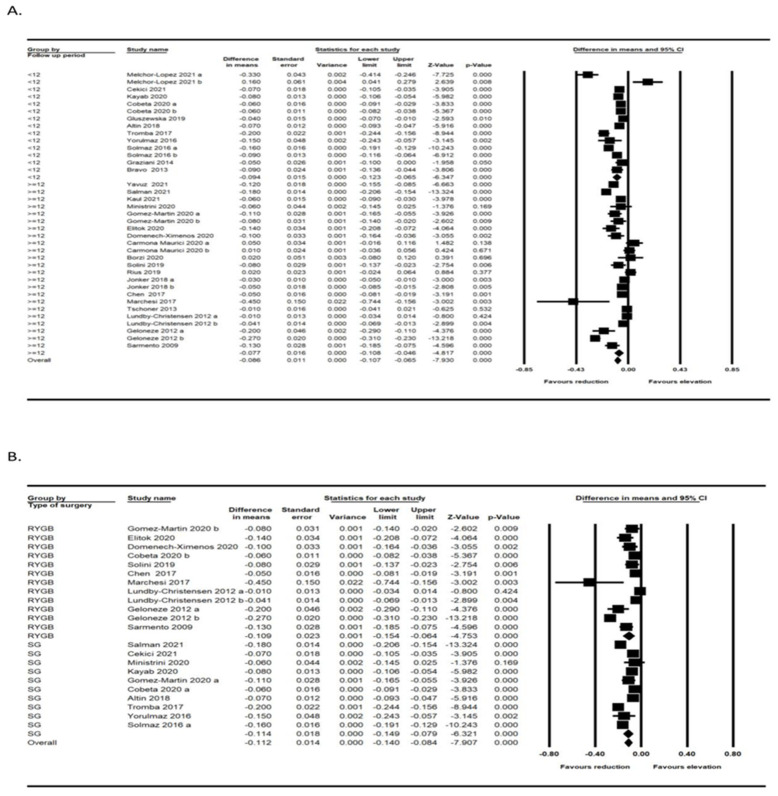
Subgroup analysis based on the follow-up period [32,33,34,35,36,37,39,40,41,42,43,45,46,47,48,49,50,51,52,53,54,55,56,57,58,59,60] (**A**) and type of surgery [33,37,38,39,40,41,42,45,49,50,52,53,54,58,59,60] (**B**).

A subgroup analysis was also performed based on surgery type and treatment duration (<12 months and ≥12 months). The subgroup analyses demonstrated significant associations between surgery types and IMT changes (*p* < 0.001). The improvement of IMT in patients who had laparoscopic sleeve gastrectomy (LSG) surgery was better than in those who had Roux-en-Y gastric bypass (RYGB). Furthermore, a significant effect of follow-up duration on the changes of IMT after bariatric surgery was observed with further improvement in IMT in the follow-up period of less than 12 months (Figure 4A,B).

### 3.5. Publication Bias

**Figure 5:**
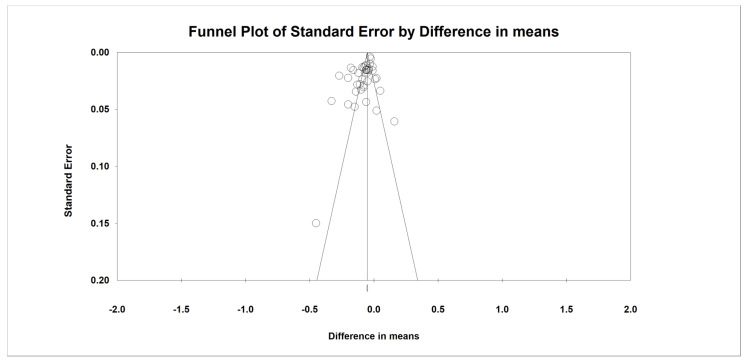
Funnel plot of standard error by difference in means.

Although the results of Egger’s linear regression test (intercept = −2.685, standard error = 0.892; 95% CI = −4.493, −0.877, t = 3.009, df = 37, two-tailed *p* = 0.004) suggested that publication bias existed in the meta-analysis concerning the effect of bariatric surgery on IMT, Begg’s rank correlation test (Kendall’s Tau with continuity correction = −0.143, z = 1.282, two-tailed *p*-value = 0.199) did not indicate the presence of publication bias. The trim and fill test showed three “missing” studies in order to adjust publication bias. The “fail-safe N” test showed that 39 missing studies would be needed to reduce the effect size to a non-significant (*p* < 0.001) level (Figure 5).

## 4. Discussion

The results of this meta-analysis of 30 trials, including 1488 subjects, showed a significant decrease in IMT after bariatric surgery, and the reduction in IMT was also robust in the leave-one-out sensitivity analysis. It must be stressed that the results of the random-effects meta-regression did not suggest any relationship between the changes in IMT and delta BMI or duration of follow-up after the bariatric surgery. However, the subgroup analyses showed significant associations between the surgery types and IMT changes and a significant effect of follow-up duration on the changes of IMT after bariatric surgery.

One important question which might theoretically influence the results of this meta-analysis is a sex difference in obese subjects concerning IMT. However, this issue was clarified in a recent study showing that IMT was significantly higher in men than in women but this difference disappeared after adjustment for covariables, such as waist circumference, age, HDL-cholesterol, and mean arterial blood pressure [61]. It has also been shown that bariatric surgery caused a significant IMT decrease in subjects with obesity in all age categories; however, the beneficial effects were more pronounced in younger individuals, which is quite understandable and easily explicable [48].

As already mentioned, it has been previously shown that obesity is associated with thicker arterial walls, i.e., increased IMT which seems to be independent of other cardiovascular risk factors [62,63]. It has been also shown that obesity is associated with T2DM and other risk factors for CHD such as dyslipidemia [64]. On the other hand, patients with severe dyslipidemia, such as familial hypercholesterolemia (FH), have increased IMT when compared with controls [65]. This is true not only for adult patients with FH but also for children with FH [66]. However, bariatric surgery in patients who had severe obesity caused a decrease in extremely atherogenic oxidized LDL particles in the blood and this phenomenon seemed to be dependent on BMI changes [10]. An earlier study showed that bariatric surgery caused a decrease in total cholesterol, triglycerides, oxidized LDL particles, and apolipoprotein B, and an increase in HDL-cholesterol and apolipoprotein A concentrations that occurred regardless of the type of surgical procedure; however, LDL-cholesterol only decreased after RYGB [67]. However, these authors could not find any correlation between the changes in serum lipid concentrations and those in IMT. A recent meta-analysis showed that pulse wave velocity (PWV) as a measure of arterial stiffness decreased significantly after bariatric surgery. This is important because atherosclerosis causes arteries to lose their elasticity and become more stiff; thereby resulting in increased PWV which predicts subsequent ACVD events [11].

Similar to our meta-analysis, a previously published small meta-analysis showed a significant reduction in IMT after bariatric surgery and indicated that the percentage of changes in BMI were associated with changes in IMT [68]. The results of the present meta-analysis also fit well with the results of another most recently published meta-analysis of 21 population-based cohort studies, involving 2,857,016 participants, which compared the effects of bariatric surgery and nonsurgical approaches on cardiovascular outcomes in patients with obesity [69]. This meta-analysis showed that bariatric surgery reduced major adverse cardiovascular events, including the risk of myocardial infarction, stroke, cardiovascular death, and all-cause death.

The present study has some limitations. Perhaps the most important one is the fact that until relatively recently there was a lack of IMT measurement standardization (method, mean/maximal thickness, carotid segment, including or excluding plaque) which could influence the predictive value in CHD risk estimation in different studies; therefore, this might also have an impact on the results of this meta-analysis [70]. In addition, according to the observational design of the included studies, we could not perform a comparative evaluation of the effects of bariatric surgery and medical treatment on IMT.

## 5. Conclusions

The results of this meta-analysis suggest that bariatric surgery significantly reduced IMT. Since increased IMT reflecting early structural atherosclerotic changes in patients with severe obesity seems to be independently associated with ACVD, the results of this study may have clinical implications for individuals with severe obesity and high cardiovascular risk. This suggests a beneficial antiatherosclerotic effect of bariatric surgery. Future prospective studies with a precise follow-up, bigger sample size, and different markers that could predict the outcomes of bariatric surgery regarding elimination of co-morbidities should add more objective data to the spectrum of benefits of weight loss surgery.

## Figures and Tables

**Table 1:** Characteristics of studies measuring IMT.

Study, Year	Study Design	Follow-up	Treatment	Control	Clinical Outcome	Patients	No. of Patients
					C-IMT		
Yavuz et al., 2021 [32]	Observational study	6 months 12 months	LSG or RYGB	-	Significant reduction was observed after 6 months	Patients with Class 3 obesity mean age of 42.3 ± 10.1 years.	41
Salman et al., 2021 [33]	Prospective study	6 months 12 months	LSG	-	Significant reduction was observed after 12 months	Patients with obesity and high cardiovascular risk M/F (63/57) mean age of 43.7 ± 8.5 years.	120
Melchor-López et al., 2021 [34]	Case–control study	9 months	LSG or RYGB	-	Significant reduction was observed No change	Patients with morbid obesity ≥ 10% reduction in CIMT Patients with morbid obesity <10% reduction in CIMT 75% F mean age 44.5 years.	28 12
Kaul et al., 2021 [35]	Prospective study	6 months 12 months	LSG or RYGB or OAGB	-	Significant reduction was observed after 6 months	Patients with obesity 70% F mean age of 40.8 ± 10.7 years.	40
Cekici et al., 2021 [36]	Prospective study	6 months	LSG	-	Significant reduction was observed	Patients with severe obesity 35F/12M mean age of 38 ± 10.48 years.	47
Ministrini et al., 2020 [37]	Single arm, open labeled, prospective pilot study	10–12 months	LSG	-	Significant reduction was observed	Patients with severe obesity 77.2% F, average age of 44.0 ± 10.1 years.	54
Kaya and Elkan, 2020 [38]	Prospective cohort study	6 months	LSG	-	Significant reduction was observed	Patients with morbid obesity 67.6% M mean age of 37.6 ± 11.2 years.	71
Gómez-Martin et al., 2020 [39]	Prospective study	12 months	LSG RYGB	diet and lifestyle modification	Significant reduction was observed in both groups compared with baseline and control group	Women with severe obesity mean age of 48 ± 9 years.	20 20
Elitok et al., 2020 [40]	Observational study	3 months 6 months 9 months 12 months	RYGB	-	Significant reduction was observed after 9 months	Patients with morbid obesity 13 F Mean age of 40.4 ± 5.6 years.	23
Domenech-Ximenos et al., 2020 [41]	Prospective observational study	3 years	RYGB	1. nonsurgical approaches 2. without any intervention (healthy controls)	Significant reduction was observed compared with nonsurgical approaches	Patients with class 3 obesity 17 F 46 (38–54) age	21
Cobeta et al., 2020 [42]	Observational study	6 months	LSG RYGB	diet and lifestyle modification	Significant reduction was observed in both groups compared with baseline and control group	Men with severe obesity and high cardiovascular risk 48 ± 8 age	20 20
Carmona-Maurici et al., 2020 [16]	Observational study	6 months 12 months	RYGB or LSG	-	No change	Patients with obesity and plaque Patients with obesity without plaque F (56%) mean age of 51.8 ± 1.8 years.	32 34
Borzi et al., 2020 [43]	Observational study	6–24 months (mean:16 ± 8)	Adjustable GB or GBP or BPD	Medical nutrition treatment	No change	Patients with obesity F/M 13/4 Mean age of 39.8 ± 10.4 years.	17
Yang et al., 2019 [44]	Retrospective study	12 months	RYGB or LSG	-	Significant reduction was observed	Patients with obesity and T2D Patients with obesity without T2D	28 62
Solini et al., 2019 [45]	Prospective observational study	12 months	RYGB	-	Significant reduction was observed	Nondiabetic subjects with severe obesity F/M 19/6 Mean age of 46.7 ± 12.9 years.	25
Gluszewska et al., 2019 [46]	Prospective cohort study	10 days 6 months	RYGB or LSG	-	Significant reduction was observed after 6 months	Patients with extreme obesity 45% M Mean age of 45.6 (±10.9) years.	71
Rius et al., 2019 [47]	Case-control study	12 months	RYGB or LSG	-	No change	Patients with morbid obesity 77.5% F Mean age of 45.0 ± 11.7 years.	33
Jonker et al., 2018 [48]	Prospective study	6 months 12 months	LSG or RYGB	-	Significant reduction was observed after 12 months	Women with obesity Men with obesity F 83.1% Mean age of 42.5 (19.4–62.1) years.	111 35
Altin et al., 2018 [49]	Prospective study	6 months	LSG	-	Significant reduction was observed	Patients with severe obesity (79F/26M) Mean age of 43.61 12.42 years.	105
Tromba et al., 2017 [50]	Observational study	3 months 6 months	LSG	-	Significant reduction was observed after 6 months	Patients with obesity 27 F Mean age of 38.7 ± 9 years	45
Chen et al., 2017 [51]	Retrospective study	12 months	RYGB	-	Significant reduction was observed	Patients with obesity and T2D F/M 17/16 Mean age of 47.7 ± 11.6 years	33
Marchesi et al., 2017 [52]	Prospective study	1 month 12 months	RYGB	-	Significant reduction was observed after 12 months	Women with morbid obesity Mean age of 42.68	22
Yorulmaz et al., 2016 [53]	Prospective study	4–5 months (average: 4.6 months)	LSG	-	Significant reduction was observed	Patients with minimum BMI of 40, who did not have any known chronic diseases 14F/2M, Average age of 39.12 ± 10.63 years.	16
Solmaz et al., 2016 [54]	Prospective study	3 months 6 months	LSG LGP	-	Significant reduction was observed after 3 months	Patients with obesity F/M 31/17 42.96 ± 7.87 (LSG) 38.3 ± 9.88 (LGP)	25 23
Graziani et al., 2014 [55]	Observational study	252 ± 108 days	bariatric surgery	-	No change	Patients with obesity Mean age of 39.8 ± 8.0	48
Tschoner et al., 2013 [56]	Prospective study	5 years	SAGB or GBP	-	Significant reduction was observed	Patients with morbid obesity 40F/12M Mean age of 35.3 years.	52
Bravo et al., 2013 [57]	Prospective study	354 ± 92.1 days	LSG or RYGB	-	Significant reduction was observed	Patients with obesity Mean age of 43.6 ± 8.1 years.	27
Lundby-Christensen et al., 2012 [58]	Observational prospective study	6 months 12 months	RYGB	-	Significant reduction was observed 12 months after RYGB in patients with T2D/IGT	Patients with obesity and normal glucose tolerance 31.3% M Mean age of 44.8 ± 10.4 Patients with obesity and type 2 diabetes or impaired glucose tolerance (T2D/IGT) 33.3% M Mean age of 47.4 ± 6.7	16 18
Geloneze et al., 2012 [59]	Observational study	1 month 6 months 12 months	RYGB	-	Significant reduction was observed	Patients with obesity without Gly482Ser polymorphism 24F/2M Mean age of 37.2 ± 10.7 Patients with obesity and Gly482Ser polymorphism of the ppargc1a gene 23F/6M Mean age of 37.2 ± 9.4	26 29
Sarmento et al., 2009 [60]	Observational study	3 months 6 months 12 months	RYGB	-	Significant reduction was observed after 6 months	Women with morbid obesity Mean age of 44.1 ± 9.8 years.	18

RYGB, Roux-en-Y Gastric Bypass; LSG, Laparoscopic Sleeve Gastrectomy; OAGB, One Anastomosis Gastric Bypass; GB, gastric banding; GBP, Gastric bypass; BPD, Biliopancreatic diversion; LGP, Laparoscopic Gastric Plication; SAGB, Single-anastomosis gastric bypass.

**Table 2:** Quality of bias assessment of the included publication in accordance with the Newcastle–Ottawa scale (cohort studies).

Study	Selection				Comparability	Outcome		
	Representativeness of the Exposed Cohort	Selection of the Nonexposed Cohort	Ascertainment of Exposure	Demonstration that Outcome of Interest Was not Present at the Beginning of the Study	Comparability of Cohorts on the Basis of the Design or Analysis	Assessment of Outcome	Follow-up Was Not Long Enough for Outcomes to Occur	Adequacy of Follow-up of Cohorts
Yavuz et al., 2021 [32]	*	-	*	*	-	*	*	-
Salman et al., 2021 [33]	*	-	*	*	-	*	*	*
Kaul et al., 2021 [35]	*	-	*	*	-	*	*	*
Cekici et al., 2021 [36]	*	-	*	*	-	*	*	*
Ministrini et al., 2020 [37]	*	-	*	*	-	*	*	*
Kaya et al., 2020 [38]	*	-	*	*	-	*	*	*
Gómez-Martin et al., 2020 [39]	*	*	*	*	*	*	*	*
Elitok et al., 2020 [40]	*	-	*	*	-	*	*	*
Domenech-Ximenos et al., 2020 [41]	*	*	*	*	*	*	*	*
Cobeta et al., 2020 [42]	*	*	*	*	*	*	*	*
Carmona-Maurici et al., 2020 [16]	*	-	*	*	-	*	*	-
Borzi et al. 2020 [43]	*	-	*	*	-	*	*	*
Yang et al., 2019 [44]	*	-	-	-	-	*	*	-
Solini et al., 2019 [45]	*	-	*	*	-	*	*	*
Gluszewska et al., 2019 [46]	*	-	*	*	-	*	*	*
Jonker et al., 2018 [48]	*	-	*	*	-	*	*	-
Altin et al., 2018 [49]	*	-	*	*	-	*	*	*
Tromba et al., 2017 [50]	*	-	*	*	-	*	*	*
Chen et al., 2017 [51]	-	*	*	-	*	*	*	*
Marchesi et al., 2017 [52]	*	-	*	*	-	*	*	*
Yorulmaz et al., 2016 [53]	*	-	*	*	-	*	*	*
Solmaz et al., 2016 [54]	*	-	*	*	-	*	*	*
Graziani et al., 2014 [55]	*	-	*	*	-	*	*	*
Tschoner et al., 2013 [56]	*	-	*	*	-	*	*	*
Bravo et al., 2013 [57]	*	-	*	*	-	*	*	*
Lundby-Christensen et al., 2012 [58]	*	*	*	*	-	*	*	*
Geloneze et al., 2012 [59]	*	-	*	*	-	*	*	*
Sarmento et al., 2009 [60]	*	-	*	*	-	*	*	*

**Table 3:** Quality of bias assessment of the included publication in accordance with the Newcastle–Ottawa scale (case-control studies).

Study	Selection				Comparability	Exposure		
	The Definition Was Adequate	Representativeness of the Cases	Selection of Controls	Definition of Controls	Comparability of Cases and Controls on the Basis of the Design or Analysis	Ascertainment of Exposure	The Same Method of Ascertainment for Cases and Controls	Non-Response Rate
Melchor-López et al., 2021 [34]	*	*	*	*	-	*	*	*
Rius et al., 2019 [47]	*	*	*	*	*	*	*	*

## Data Availability

Not applicable.
